# Two Routes to Losing One’s Past Life: A Brain Trauma, an Emotional Trauma

**DOI:** 10.3233/BEN-2008-0213

**Published:** 2009-06-01

**Authors:** Julie Ouellet, Isabelle Rouleau, Raymonde Labrecque, Gilles Bernier, Peter B. Scherzer

**Affiliations:** ^1^Department of PsychologyUniversité du Québec à MontréalMontréalQCCanada; ^2^Neurology ServiceNotre-Dame HospitalCentre Hospitalier de l'Université de Montréal (CHUM)MontréalQCCanada

**Keywords:** Retrograde amnesia, remote memory loss, psychogenic origin, functional retrograde amnesia, autobiographic amnesia

## Abstract

Organic and psychogenic retrograde amnesia have long been considered as distinct entities and as such, studied separately. However, patterns of neuropsychological impairments in organic and psychogenic amnesia can bear interesting resemblances despite different aetiologies. In this paper, two cases with profound, selective and permanent retrograde amnesia are presented, one of an apparent organic origin and the other with an apparent psychogenic cause. The first case, DD, lost his memory after focal brain injury from a nail gun to the right temporal lobe. The second case, AC, lost her memory in the context of intense psychological suffering. In both cases, pre-morbid autobiographical memory for people, places and events was lost, and no feeling of familiarity was experienced during relearning. In addition, they both lost some semantic knowledge acquired prior to the onset of the amnesia. This contrasts with the preservation of complex motor skills without any awareness of having learned them. Both DD and AC showed mild deficits on memory tests but neither presented any anterograde amnesia. The paradox of these cases–opposite causes yet similar clinical profile–exemplifies the hypothesis that organic and psychogenic amnesia may be two expressions of the same faulty mechanism in the neural circuitry.

## 1. Introduction

Retrograde amnesia (RA) refers to the inability to recall information acquired prior to the onset of a cerebral pathology. Up until a few years ago, this amnesic syndrome was neglected by memory researchers in favour of anterograde amnesia study, which is easier for caregivers to identify, more accessible to measurement for clinicians and more disabling for patients [16]. However, in recent years there have been a growing number of studies on RA, more specifically on a subgroup of memory disturbance, *focal* retrograde amnesia (FRA), whose existence is still a matter of debate in light of the existing models of long-term memory. The core feature of FRA is a severe loss of retrograde memory (principally episodic), combined with largely preserved anterograde memory ability [12]. In 1885, Ribot (cited by Kapur [14]) anecdotally reported a case, which he described as "an example of incurable amnesia, extending only onto the past". Since then, patients showing this syndrome have been described several times in modern literature. These cases can be roughly divided into two broad groups [17]. On the one hand, there are those who have lost their memory after a neurological disease. On the other hand, there are patients with FRA who do not show detectable neurological dysfunction but for whom a psychogenic aetiology is presumed to be at the source of the memory disturbance. This organic-psychogenic dichotomy has been clouded by a considerable number of cases whose organic and/or psychological aetiology remains unclear. An example of such a confusion would be a patient with some pre-morbid psychosocial difficulties who suffers a mild concussion and becomes amnesic of part or the whole

ISSN 0953-4180/08/$17.00 © 2008 – IOS Press and the authors. All rights reserved of his autobiographical past, showing a RA which is very much disproportionate to what would be expected on the basis of the cerebral pathology alone([10,18,23, 39], and for a review, see [34]).

A wide variety of brain lesions and metabolic deficiencies have been associated with FRA (for a review, see [14,16]). They range from brain injury, herpes simplex encephalitis and epilepsy to vascular pathology and hypoxia. Diffuse rather than focal lesions are known to produce FRA. When the damage is circumscribed it is most commonly bilateral [27], and there is always some direct or indirect involvement of the temporal lobes [20]. An exception to this observation is the description by Repetto et al. [36], of a patient with focal transient hypometabolism in the right globus pallidus and putamen and persistent significant autobiographical semantic and episodic memory gaps 9 months after the single event. The semiology of organic FRA may or may not be characterized by a temporal gradient, the memory deficit usually encompasses autobiographical data and public events and faces, but can also cover encyclopaedic knowledge.

Retrograde amnesia following a stressful or traumatic event has intrigued authors since the turn of the $19^{th}$ century. More recently, psychoanalytic psychiatrists coined the term "dissociative amnesia" to denote the phenomenon. Amnesic symptoms in these patients usually range from gaps in memory lasting from minutes to days, and the memory loss usually ends within a few hours or days after its initial presentation [25]. Individuals with psychogenic amnesia most typically only lose access to their personal data, although semantic knowledge may be affected as well [18].

Despite the fact that organic and psychogenic amnesia can be quite similar in their presentation, few links have been drawn between them until recently. Previously organic amnesia was a domain of interest for neurologists whereas psychogenic amnesia was an area of interest for psychiatrists, with few overlaps between the two fields. An attempt to reach a unified comprehension of the two syndromes has recently come from neuropsychology. Its growing diagnostic sophistication and contributions to the conceptual understanding of neurological diseases and its more recent contribution in the field of psychiatry have led many authors to discuss similarities and differences between organic and psychogenic amnesia. Markowitsch [25] and Kopelman [18] particularly, have argued that these classifications represent "two sides of the same coin", or form end-points along a continuum rather than discrete categories, and that organic and psychogenic amnesia may stem from common pathophysiological mechanisms. Lying within this framework of ideas, the present paper is, to our knowledge, the first to directly compare two patients suffering from a strikingly similar dense and focal retrograde amnesia, despite their two apparent different aetiologies.

## 2. Material and methods: Case reports

### *2.1. Case AC: Psychogenic amnesia*

AC, a 39-year-old right-handed women, was brought to the hospital emergency on April $11^{th}$ 1998, a few moments after having attempted suicide, threatening to jump from a window. She was stopped from doing so by her boyfriend. On arrival of the ambulance, AC was well oriented in time and place. Upon examination in the emergency unit, she was described as being excessively anxious and pacing like a lion in a cage. Her contact with reality was adequate; she expressed feelings of emptiness, loneliness and fear of loosing control. She was given medication for her anxiety and was released from the hospital the same day. Two days later, she appeared to have lost all memory of her previous life. Neurological examination and a head computerized tomography performed at that moment were unremarkable. However, AC could not recall anything pertaining to her past. She did not know her own name, address, nor did she remember the suicide attempt of two days previously nor the reasons that had led her to the attempt. She did not know whether she had a family of her own, if her parents were dead or alive nor how long she had been living in this city. On the other hand, memory for on-going events was clinically normal.

We could not get many details directly from AC concerning the four to six months following the loss of her memory, as this period remains too vague for the patient. According to medical records, the following months were marked by an addiction to Rivotril and alcohol, as well as intense psychological suffering mostly due to an abusive relationship with her boyfriend and financial distress.

Six months after the onset of the amnesia, AC commenced weekly psychotherapy sessions with a psychiatrist. She did not undergo hypnosis nor were there any Amytal injections. Following her psychiatrist's advice, she paid special attention to her dreams, hoping that some elements of her past would emerge from them. She reports having experienced at that time a great deal of frustration and helplessness as a result of not being able to recall anything. According to AC, neither friends nor the boyfriend had reported her memory loss to her family, and it was left to the psychiatrist to do so. He eventually contacted her ex-husband of fifteen years, who appeared on some of her documents as being the person to contact in case of emergency. AC did not know until then that she had been previously married.

Subsequently, the ex-husband re-established a relationship with AC and was instrumental in helping her to reconstruct her past. He showed her pictures, told her about their life together and about their three children (aged between 20 and 25 at the time). He made frequent trips between the city where he lived and her home to ensure that she met with her children as often as possible. On the first of these reunions, she did not recognize her children, nor did she experience any sense of familiarity towards them. She also did not recognize her mother and sister when she met with them after the onset of her amnesia, and she called her mother "Ma'am" for a period of time during the subsequent family meetings. A visit to the city where she raised her children and lived for 15 years did not evoke any memories. Looking at her photographs from childhood to marriage did not evoke any memories either.

We interviewed separately the two people who seem to have known her the best: her sister and her ex-husband, in order to better comprehend AC's present condition and to fill in the many gaps in her information about her past. AC left home at 14 because of major disagreement with her father, and possibly because of domestic violence. She had her first child at 15 and two other children before 20. She is described as having always been a hardworking, intelligent women, although she had been struggling with a drinking problem ever since she was a teenager. Both AC and her husband at the time had been drinking quite heavily for many years, and this situation caused them to lose custody of their children for a few years. After the divorce, AC showed many signs of depression and suicidal tendencies, symptoms that culminated when she entered a three-year relationship with an abusive man – the same man she was living with when she lost her memory.

### 2.2. Past and present psychiatric status

Information was collected through the patient's medical record. Five years prior to the onset of amnesia, AC was diagnosed with a severe panic disorder and depression, two conditions that have persisted throughout the following years and past the onset of the amnesic syndrome. She is also known to have made a few attempts at suicide during the same period. After she lost memory, AC was diagnosed with a "Dissociative amnesia with depressive-anxiety problems on axis 1 and marital difficulties including violence that triggered the amnesia on axis V". Hysterical and obsessive-compulsive traits were also noted in the patient. Dual or multiple personalities disorder diagnostic were considered and dismissed by the psychiatrist. According to family members, AC has no known past history of fugue episodes.

We had AC fill out a Millon Clinical Multiaxial Inventory (MCMI-revised). The profile could not be validly interpreted since a total of 26 questions, corresponding to elements pertaining to the past, were left unanswered.

### 2.3. Clinical observations

We met AC four, then five years (43 then 44 years old) after the onset of her amnesia. She was referred by her neurologist whom she had consulted twice during the previous year for persistent headaches and learning difficulties she was experiencing in a one-year secretary course. These are the only known neurological antecedents in AC.

AC is a slim, tall woman who looks her age. On each of the six occasions we met her, she was timid, quiet and somewhat serious. Her general attitude indicates that she was still upset by her condition. In interviews, she often looked perplexed and sometimes sad when she could not provide answers about her past. AC reported that most of her time and energy was occupied by trying to find her "past self" for the two years following the onset of the amnesia. Even though she finds it hard to remain with the uncertainty about who she was and what kind of life she had been leading up until her memory loss, she has now decided to get on with her present life and plan her future. She was an eager collaborator throughout the process of collecting information for the present investigation.

### 2.4. Neuroradiological examination

In addition to the negative CT scan performed after the onset of AC's memory disturbance, a MRI and a resting state SPECT were done, five years post-trauma. The MRI did not show any sign of structural brain damage and the SPECT did not show any focal region of hypometabolism..

## 3. Neuropsychological assessment

### *3.1. Anterograde memory*

The patient's anterograde memory was assessed with standard learning tests and semi-structured interviews. On a clinical basis, AC's memory for day-to-day events appears intact, as she is well able to recall events that had occurred in the five years "post-trauma". Although she is able to keep track of incoming information, she complains of experiencing some learning difficulties at school. Previously a "straight A" student, according to transcripts of marks from the early 90s, when she went back to school as an adult student, she now reports having many difficulties keeping pace with her fellow students in a secretarial school for adults.

Results of the formal testing on memory measures are shown in Table 1. We tested AC's verbal memory in both English and French, in order to determine whether her difficulties in secretarial school were a consequence of the teaching language (French), which is less familiar to her, or to verbal encoding deficits *per se*. AC was raised in French and spoke French fluently until the age of 14. She eventually lost some of her abilities in that language having switched to English when she met her husband. She raised her children in English.

AC shows a mild encoding deficit for words in the Rey Auditory Verbal Learning Test (RAVLT), administered in French, but not in the Logical stories or the Verbal Paired Associates of the Weschler Memory Scale III, administered in English. The learning slope is a bit flat on the RAVLT and some retroactive interference is noted. These difficulties, and those encountered at school, can be attributable to her loss of familiarity with the French language. On the other hand, her visual memory seemed completely normal as measured by the different tests (WMS-III and Rey-O Figure).

### *3.2. Retrograde memory*

#### *3.2.1. Autobiographical memory*

Autobiographical memory for AC was investigated with the *Autobiographical Memory Interview* (*AMI*) [22] and with a semi-structured interview. The AMI contains questions about personal semantic (*Name of first school*) and autobiographical incidents (*Recall an incident from the first job*) for three periods of the patient's life: Childhood, Early Adult Life and Recent Life. AC was unable to recollect any single personal incident in the Childhood or Early Adult Life sections of the AMI. On the other hand, she could easily talk about the last five years of her life. No personal semantic information was recalled by AC concerning her childhood, whereas she could recount a few details concerning her early adult life since some relearning had occurred (qualifications obtained after leaving school, name of first child). Her total score in the Personal Semantic Information section was 30/63, and she scored 9/27 on the Autobiographical Incidents section. During interviews, AC always denied having any memory of her entire life history prior to April 1998. The only so-called memory she had that predated her amnesia was the feeling of having been pregnant. All the other facts she knew pertaining to her past (birth year of her children, previous occupations, year of marriage, etc.) had been relearned in a detached manner through conversations she had had with family members and her ex-husband. Those "memories" are totally without any emotions or sense of intimacy.

#### *3.2.2. Semantic memory*

Semantic information that had been learned before 1998 seems to have been only partly preserved, although this is hard to rigorously assess four years post-onset since a relearning of many facts may have taken place. AC did lose all explicit knowledge of a nine-month class she took in the mid 90s in health-care aid-work, about three years prior to the onset of her amnesia. This program included a computer class. At the time of the assessment by our group, AC was taking a one-year class to become a secretary – a course that probably duplicated some of the things she had learned pre-trauma. She mentioned that some of the apprenticeships did give her a feeling of déjà-vu, but this never evolved into an explicit recall. The loss of semantic memory extended to the French language. Medical notes dating back to the months following her loss of memory indicate that she could not properly answer questions in French. AC reported later to her psychiatrist that she did understand the language, but she was unaware that she once was able to speak it until her ex-husband told her so. She eventually relearned the language, but not without difficulty.

AC also seemed to exhibit a partial loss of didactic information that was still evident four years post-onset. The score on the Information subtest of the WAIS-III was the lowest score of the intelligence scale (scaled score = 5). Some responses to this subtest were particularly surprising given her level of education (13 years). For example, she had no idea when asked "*On what continent is the Sahara desert?*". She did not know what a continent was and examples provided did not

Table 1
DD and AC's scores on standard anterograde memory tests

| | DD Raw data | Z score/%ile Scaled score (ss) | AC Raw data | Z score/%ile Scaled score (ss) |
|---|---|---|---|---|
| Verbal memory | | | | |
| RAVLT | | | | |
| Trial 1–5 | 8-9-11-12-12 | | 5-8-8-7-11 | |
| Total words recalled | 52 | z = 0.74 | 39 | z = −1.63 |
| Immediate recall | 10 | z = 0.36 | 8 | z = −0.53 |
| 30-minute delayed recall | 13 | z = 1.43 | 9 | z = −0.12 |
| Immediate recognition | 15/0 false rec. | n/a | 13/0 false rec. | n/a |
| Delayed recognition | 15/ false rec. | z = 2.10 | 13/0 false rec. | z = 0.31 |
| Logical memory | (WMS-R) | | (WMS-III) | |
| Immediate recall | 18/50 | ss = 7 | 27/75 | ss = 6 |
| Delayed recall (scaled scores) | 11/50 | ss = 7 | 10/50 | ss = 8 |
| Visual memory | | | | |
| Rey-Osterrieth figure | | | | |
| Immediate recall | 11.5 | $5^{th}$ % ile | 19 | $31^{th}$ % ile |
| Delayed recall | 10.5 | $3^{th}$ % ile | 18 | $24^{th}$ % ile |
| Visual memory | | | | |
| Visual Reproduction | (WMS) | | (WMS-III) | |
| Immediate recall | 6/15 | z = −1.05 | 94/104 | ss = 13 |
| Delayed recall | 4/15 | z = −1.36 | 59/104 | ss = 9 |
| Recognition | 1/4 | n/a | 42/48 | ss = 8 |
| Face recognition | Warrington | | Faces (WMS-III) | |
| | 43/50 | ss = 9 | Immediate = 30 | ss = 7 |
| | | | Delayed = 35 | ss = 9 |

Z scores: measure of deviation from the mean (mean = 0, standard deviation = 1); normal range from −1 to 1. Ss: scaled scores established on normative data (mean 10, standard deviation = 3); normal range from 7 to 13. %ile: percentile rank established on normative data (lowest 0, highest 100); cut-off score: 16th % ile. n/a: norms are not available for these tests.

help her grasp the concept. She answered "*France*" to the question "*What is the capital of Italy?*", and had no idea of who Cleopatra was. It is possible, however, that part of this loss might be explained by a limited academic achievement. It must also be recalled that CC left her parents' home at 14 and had her first child when she was 15. Performance on Vocabulary was in the superior mean range (scaled score: 12).

AC's explicit recall of famous names was also tested with a test that covers six decades [42]. The overall performance was below that of control subjects and there was no evidence of a temporal gradient, although the patient was more successful with items covering the last decade (78% correct responses for the 1990's, compared to 62–64% correct responses for the 1960's, 70's and 80's).

### *3.2.3. Procedural memory*

AC shows preservation of at least one complex motor skill that was learned before the onset of amnesia: typewriting. When in her first computer class two years post-trauma, she was surprised to realize that she knew where to place her fingers on the keyboard and was quite fast at typing. She did not however, have any recollection of how she learned this skill.

## *3.3. Other cognitive functions*

An exhaustive neuropsychological assessment was also conducted with AC. Language abilities were normal, except for a slight deficit in confrontation naming for low frequency words. Perceptual functions were in the normal range. Attentional capacities (simple immediate span of attention, sustained and focused attention) all appeared normal as well. Upon examination of executive functions, AC showed normal planning abilities on the Tower of London, WISC Mazes, Zoo Map and Modified Six Elements Test (BADS). However, moderate difficulties were encountered on the copy of the Rey-Osterrieth Figure. The poor reproduction of the figure (result was 27/36) appears to be the product of haste and carelessness rather than a consequence of visual-spatial or executive disorder. The Wechsler Adult Intelligence Scale (WAIS-III: Short form [35]) indicates that AC was functioning within the average range of intelligence, according to estimates of VIQ = 85, PIQ = 94 and FSIQ = 88 [35].

## *3.4. Case DD: Organic amnesia*

DD was a 40-year-old, right-handed man who, before his accident, was without any previous medical, surgical or psychiatric history. On November 29 1977, while doing renovations at his workplace, a nail from a hydraulic gun entered his right temporal region. The patient was found somnolent on the accident site but was conscious upon arrival at the hospital and responded adequately to verbal commands. The neurological exam revealed a left visual field defect, a left facial paresis, a left hemiparesis with positive Babinski sign, and a left hypoesthesia to all modalities. He was immediately transferred to neurosurgery: a right lateral craniotomy was performed and a 3 cm-long nail was removed.

Post-operative evolution was considered favourable. The patient spent three weeks at the hospital recuperating from the accident and the surgery, with his wife by his side. Although he was medicated and sedated for much of the time during this period, he recognised that he was in a hospital and he kept asking his wife and the medical staff why he was there. He was discharged 24 days following admission. Only when he returned home did it become obvious that DD had lost his past memories.

At the time of the accident, the patient was married and the father of two sons, aged 14 and 16. He was the manager of an electronics firm where he had been employed for 20 years. After a six-month convalescence period the patient went back to work although he still presented a left sensory-motor deficit that was associated with a painful sensation (thalamic syndrome). He had to relearn most of the information pertaining to his work (names and occupations of his colleagues, clients and suppliers, how to make a contract, etc.). He needed frequent rest periods during the day because of his fatigability. He was only able to succeed with the constant help of his wife and support of his colleagues. In 1985, seven years after his accident, the company was sold and his work re-evaluated by the new owners. His performance as a manager was then judged unsatisfactory and he was offered a salesman position, which he refused. This demotion greatly affected the patient psychologically and apparently exacerbated his chronic pain. He has since been considered unfit to work but has remained active in voluntary work.

In 1988, eleven years post-trauma, the patient had a comprehensive workup (neurology, neuroradiology, neuropsychology, clinical psychology and psychiatry) prior to the implantation of a thalamo-lemniscal electrode for the treatment of his pain syndrome. The neurologic exam was essentially unchanged. It revealed a subtle hemiparesis with increased tendon reflexes and positive Babinski sign on the left, a left hemihypoesthesia to all modalities, athetoid movements of left hand with coarse postural tremor, a dysmetria of the left hand and foot on cerebellar testing and a left inferior quadranopia. The patient complained of recurrent headaches that were partially controlled with medication. According to the CT- scan (August 1985) and to the MRI scan performed in May 1989 before the implantation of the electrode, the lesion was unilateral and linear and extended from the mid portion of the right superior temporal gyrus to the thalamus (VPL, pulvinar and possibly CM). The lesion involved the posterior part of the insula, the extreme capsule, the claustrum and part of the temporal stem. The mammillo-thalamic tract, amygdala, hippocampus, entorhinal, parahippocampal and cingulate gyri appeared intact as did as the entire left cerebral hemisphere (see Fig. 1a, b).

### *3.5. Past and present psychiatric status*

Information was collected through the patient's medical records and interviews with his wife in 1988, and from formal psychological and psychiatric assessment. Past psychiatric history was unremarkable. According to his wife, DD had never shown any signs of psychiatric illness in the past. He completed 14 years of formal education with specialization as an electronic technician. He married when he was 21. The couple had two sons, one of whom was diagnosed with schizophrenia seven years after DD's accident. DD has been involved as a volunteer in an association for parents of schizophrenic children since his son's diagnosis. Before his accident, DD seemed fulfilled by his work and his family life. He always maintained close relationships with his brothers, sisters and children. Other than his actual condition, DD is and has always been in good health. He drinks only socially.

Results of the MMPI are considered invalid since a total of 52 questions pertaining to the patient's past were left unanswered. Results obtained on the Beck Depression Inventory and on the ASTA in 1988 showed mild levels of anxiety and depression. Psychiatric evaluation revealed a neutral affect with some depressive elements. The patient has no post-traumatic stress disorder symptoms or conscious emotional distress related to his condition. According to his wife, the patient had not changed after his accident as far as personality, tastes and preferences (food, clothing, music, etc.) were concerned.

The patient was briefly re-interviewed in December 1993. Following our 1988 investigation, a thalamo-

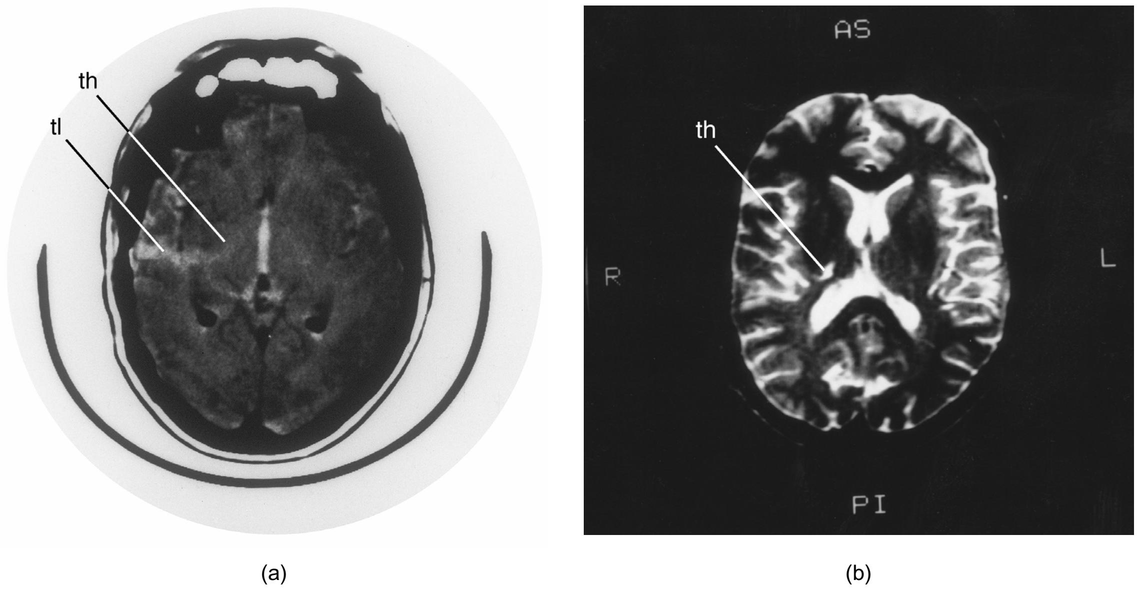

Fig. 1. CT-scan (a) and MRI T2 weighted scan (b) showing a right unilateral lesion in DD extending from the mid portion of the right superior temporal gyrus (indicated as tl) to the thalamus (indicated as th).

lemniscal electrode was implanted for pain control but it had to be removed a year later because of infection, even though it did alleviate some of the pain. Treatment with hypnosis and acupuncture later brought him some relief but he was still taking medication. DD's wife died from cancer in May 1993. Although their relationship was particularly close because his wife was, in a way, his autobiographical memory, he completed the mourning process by participating for a few months in a support group for widowers. He is now leading a normal, active life as a volunteer worker and he is living by himself. He still does not "remember" anything of his autobiography for the period prior to his accident in 1977.

## 4. Neuropsychological assessment

### *4.1. Anterograde memory*

With the exception of difficulty in recalling the early postoperative period, the patient presented no anterograde amnesia and could adequately recall ongoing events. Contrary to what was noted for events preceding his accident, his mental imagery of events that occurred after the trauma was normal. However, some deficits were apparent on formal testing of memory functions. The scores obtained on memory tests are presented in Table 1. Results obtained with verbal material (RAVLT, WMS-R) were better than those obtained with visual-spatial material (Rey-Osterreith, WMS-R), although some retrieval deficits were observed in the delayed recall of Logical Memory and Verbal Paired Associates. Significant improvement in performance was seen when retention was assessed with recognition. No evidence of malingering was observed ; the patient obtained a score of 98.75% correct in a forced-choice picture recognition test known to be sensitive to malingering [5].

### *4.2. Retrograde memory*

Although the patient was evaluated more than ten years post-trauma, his well preserved anterograde memory for the entire period following the onset of his illness allowed us to analyze the characteristics of his retrograde memory loss.

#### *4.2.1. Autobiographical memory*

Autobiographical memory was assessed with semi-structured interview with the patient and his wife in 1988. It was reported that when he was released from the hospital and back at home with his wife and children, it became evident that DD had completely lost his autobiographical memory. He could not remember any single personal events for his entire life (first year of school, wedding, birth of his children, etc.). He did not recognize his house although he had built most of the interior himself, nor the office where he had worked for 20 years. He did not recognize personal belongings. He could not recognize his relatives, colleagues or long-time friends. He experienced no sense of familiarity when he visited the house in which he was raised or when looking at pictures of his parents or of himself at an earlier age. He could not remember any trip he had made in the past even when looking at pictures and did not recognize anything when he re-visited the same places. Although his well-preserved anterograde memory enabled him to relearn a lot of information about his life, he has never experienced any sense of familiarity when relearning his autobiography. He knows that when he was 32, his father died from a heart attack at age 69. DD does not recall whether his father died in his house or at the hospital, although he took care of him every day during the weeks preceding his father's death.

The episodic memory deficit extended to public events and famous people the patient should have known from reading and watching the news (confirmed by his wife). Although DD was able to relearn a number of important events that had occurred during his lifetime, he presented unexpected "gaps" in his knowledge of public events and famous people. Because there is no standardized remote memory test available for the Quebec francophone population, this last feature was assessed clinically by the presentation of pictures of famous people and recalling of their names with cueing and/or recognition if necessary, and the discussion of important public events. Although the patient had some knowledge of certain events, he could not give a detailed account since he did not "remember" them i.e., he experienced no sense of having witnessed their occurrence during his lifetime. Similarly, when presented with musical themes of famous television programs, the patient could only recognize the more recent ones, all broadcast after his accident.

#### *4.2.2. Semantic memory*

The patient reported that he had lost a great amount of previously acquired information (e.g. geography, history, literature, music), a fact that was confirmed by his wife. He had to completely relearn the map of his hometown. He could not explicitly remember anything concerning his specialized training as an electronics technician. Despite having relearned a lot of information at the time of the assessment, formal tests of general knowledge confirmed the presence of numerous gaps compared to what one would expected given his socio-cultural status and educational level. DD could not name the continents, did not know who discovered Canada, and thought Julius Caesar was a singer. His performance was not facilitated with multiple choice. He obtained a scaled score of 6 on the Vocabulary subtest. He could not provide the definition of a number of words he should have known given his premorbid level. These were low frequency words that were probably learned at an older age (e.g., reluctant, remorse, tangible, etc.). Although he did not present any obvious language deficits and his basic reading and writing skills were preserved, some grammatical rules were lost and a number of orthographic errors were made when writing dictation. Again, there errors concerned mostly low frequency words. Arithmetic skills were preserved but part of the multiplication tables had to be relearned. This was easily done and, at the time the evaluation was performed, DD did not present any difficulties solving arithmetic problems (WAIS-R scaled score: 10)

#### *4.2.3. Procedural memory*

The patient seemed to have retained a number of premorbidly acquired skills, such as driving his car, skiing, swimming and skating. This normal performance contrasted with the absence of a conscious recollection of having done those things in the past. His abilities to use tools appeared intact at least for basic operations; more complex skills could not be assessed, as the patient still presented a left sensory-motor deficit precluding the use of many tools. He had however retained certain highly complex skills such as repairing electronic circuits. As the patients spontaneously stated: "For manual things, I do it but cannot explain how... I do it without knowing why... It's in me".

### *4.3. Other cognitive functions*

A comprehensive neuropsychological assessment was conducted in DD, as part of the pre-operative workup. Confrontation naming was mildly deficient but mostly for low-frequency words. Reading was slow but no errors were made on the Chapman-Cook test. Verbal fluency was limited. As described before, DD made many errors when writing to dictation and complained of having lost many grammatical rules, a fact that was confirmed by his wife. Spelling was as deficient as was writing to dictation. Repetition was normal. A mild to moderate deficit of visuoperceptual functions was apparent on Benton's tests of Facial Recognition and Judgment of Line Orientation, where DD obtained scores of 43/54 and 21/30 respectively, and on the Hooper Visual Organisation

Test (22.5/30). There was a more severe deficit on the copy of the Rey-Osterrieth Figure, where performance was below the first percentile (23/36). On Object Assembly and Block Design, the performance was slow but normal overall. Visual exploration was slow but there was no omission on Letter Cancellation. The IQ scores were in the normal range with a slightly lower score on the Performance Scale compared to that of the Verbal Scale, consistent with DD's visual-perceptual deficits and slowing (VIQ = 92; PIQ = 85; FSIQ = 88). There was no frontal lobe semiology as assessed by WCST, Trail A & B, Luria's graphic series and gestual sequences. On the Stroop Test, the performance was slow but there was no interference effect on the third condition (name color print).

Table 2 highlights the similarities between CC and AC in terms of the general characteristics of their amnesia and memory systems.

## 5. Discussion

The two patients presented share a similar memory profile in many regards. The retrograde amnesia covers 40 years without any temporal gradient modulating the memory loss. Although both patients were able to relearn many aspects of their personal history, neither of them ever recovered the vividness and authenticity of their past memories. Indeed, not a single autobiographical memory has emerged for AC and DD since the occurrence of their respective trauma. Semantic memory also appears similarly affected in both patients, who exhibit "patchy" didactic knowledge. Furthermore, both patients showed a preservation of most motor skills acquired before the onset of the amnesia, without any memories of the acquisition of those abilities. Finally, anterograde memory functions appear mildly diminished for both patients on tests but not on a day-to-day basis. AC shows some learning difficulties for verbal material whereas DD has encoding deficits with visual stimuli, a fact that is compatible with his right-sided brain lesion.

One of the interesting observations to come out of these cases is how an organic and a psychological condition can both trigger the same profile of complete FRA. These case presentations tend to support the hypothesis of a disruption of retrieval processes rather than a deterioration of the memory substrata *per se*. The disconnection may be a consequence of mechanical alterations in the brain of organic amnesia. In psychogenic amnesia the disconnection may be induced during processing of autobiographical information in a way that can be described as a desynchronization of the activity that give access to memory patterns [25]. The similarities of the two cases provide support for the hypothesis postulating a dysfunction in a common underlying pathway in both types of amnesia. Markowitsch et al. [28] and Costello et al. [6] produced PET evidence of defective right frontal retrieval mechanisms in psychogenic and organic amnesia, a procedure that could not be used with our patients owing to their possible relearning of autobiographical information.

Taken separately, the semiology of our two patients' conditions does not match the typical features of organic and psychogenic amnesia. AC's amnesia differs from what is usually observed in psychogenic amnesia in its seven years of persistence despite a strong desire to recuperate. This is a far longer duration than what is usually observed in psychogenic amnesia which abruptly resolves within a few hours or days after its initial presentation, following a confrontation with elements of past life [1,3,25,38].

As concerns DD, he was left with a retrograde amnesia which appeared to be unusually severe in comparison to the extent of his lesion. As Teuber reported [40], he found "no case in which RA appeared in isolation" in 100 cases of penetrating brain injury, a finding also reported by Salazar et al. [37]. In general, focal retrograde amnesia is found following diffuse and bilateral lesions. Based on the existing written evidence, Kapur [15] concluded that a focal pathology of the temporal lobes is unlikely to provoke a global autobiographic retrograde amnesia. Two years later, Markowitsch et al. [27] suggest that circumscribed *bilateral* lesions can produce such an amnesia, although the precise location of the lesion remains obscure. DD's case, featuring a focal and unilateral penetrating lesion, appears to challenge these affirmations and offers a potential region of interest. The lack of anterograde amnesia in DD is consistent with preservation of the medial temporal lobe structures namely the hippocampus, entorhinal cortex and amygdala. Indeed, when penetrating head injury disrupts anterograde memory functions, it is mainly due to poor retrieval and poor control of learning rather than storage deficit [32].

An important point to discuss is the genuineness of AC's amnesia [13,19]. We believe that in the case of AC there is evidence of an authentic, unconsciously triggered hysterical amnesia. First, there is a lack of secondary gains. AC was never involved in litigation nor did she gain any financial advantages in this situation. It might be argued that AC simulated to escape her

Table 2
Differences and similarities between DD and AC's profiles

| | DD | AC |
|---|---|---|
| Origin of amnesia | Penetrating wound from a nail gun | Marital difficulties and suicide attempt |
| Age of onset of amnesia | 40 years old | 39 years old |
| Duration of amnesia at time of testing | 10 years | 5 years |
| Density of amnesia | Entire pre-traumatic life | Entire pre-traumatic life |
| Extent of lesion | Unilateral & linear, extending from superior temporal gyrus to thalamus | None found (MRI) |
| Psychiatric antecedents | None | None |
| Post-traumatic personality | Unchanged | Some changes observed: previously a go-getter, now more timid and self-effacing |
| | Investigation of Memory Systems (structured clinical interview and questionnaires) | |
| Retrograde autobiographic memory | Non-existent for persons, places and events. No feeling of familiarity during relearning of personal information. DD did not recognize his family nor the home that he had built himself. AC did not recognize her 3 children in their 20's nor her mother. | |
| Anterograde memory | No anterograde amnesic syndrome, although there was a slight difficulty learning new material (see Table 1) | |
| Semantic memory | Partial loss of didactic (geography and history) and general cultural knowledge | |
| Procedural memory | Preserved, although there was no recollection of the original learning experience for skills (e.g. capacity to repair electric circuits for DD and knowledge of typing for AC) | |

painful marital situation. However, a counter-argument is that there was no alleviation of the symptoms despite the fact that her relationship was terminated four years previously. If AC's case was one of malingering in order to get attention from the medical community, one would have expected her to get help after the onset of the amnesia. Instead, she only consulted a psychiatrist six months after her loss of memory. Also, she never mentioned anything about her amnesia to her neurologist on any of her appointments, two years post-onset. Gaining the sympathy of her relatives might also have motivated AC to simulate, but again, if that was the case, one would think that she would have found a way to alert the family about her condition earlier than six months after the onset. Furthermore, it has been reported by family members that AC did not seem comfortable nor content in family meetings following the onset of amnesia.

Second, no major inconsistencies were found between various interviews with AC and between her statements and her ex-husband's nor her sister's statements. Moreover, these two relatives were both very adamant about AC's amnesia. Third, her state of anxiety and preoccupation concerning her memory loss does not seem typical of a simulated amnesia. Lastly, the long duration of AC's amnesia (seven years) speaks in favour of a non-simulated condition, although a prolonged duration (one year) of malingering in a case study of amnesia, has been reported by Kopelman [19].

Are AC and DD valid examples of psychogenic and organic amnesia? In the case of AC, neuroradiological exams :CT, MRI and a resting state SPECT – the latter having been described as being more efficient at localizing brain dysfunction than is EEG [29] – showed no evidence of anomalies. However, this absence of physical proof does not necessarily qualify AC's syndrome as being psychogenic. A psychogenic interpretation should rest on positive history, not only on the absence of demonstrable organic factors [7,8]. In AC's case, her three-year abusive relationship and her psychiatric history (depression, panic disorder, suicidal attempts, hysterical and obsessive-compulsive traits) give stronger support to a psychogenic than neurogenic hypothesis. Furthermore, psychogenic amnesia may occur as a protective mechanism in persons contemplating suicide and psychogenic loss of memory acts as an alternative to suicide in some patients [11,41]. AC's loss of memory occurring some 48 hours after an incomplete suicide attempt appears to exemplify this view. Insofar as AC's drinking habits are concerned, this information does not likely have any etiological relevance on the grounds that there is general agreement that even long-term alcohol abuse does not result in isolated RA [4].

The evidence to date leads us to postulate that AC's social environment, psychiatric antecedents and prolonged states of altered mood may have combined to modulate her cerebral activity in a way that caused permanent change in the network dedicated to the retrieval of autobiographical memories. Although not yet well understood, a wide range of effects on memory functions and on brain regions involved in memory have been attributed to stress [3]. Also, studies showing neurobiological correlates of the effects of psychotherapy reinforce the idea that context and cognitive behavior can functionally "rewire" the brain [33].

We see a number of arguments in support of the conclusion that DD's amnesia is of neurological origin. In his case, there is an incontestable brain insult and a perfect contiguity. Indeed, his memory loss followed immediately after a focal lesion in the right hemisphere, which is more frequently involved in the retrieval of episodic information than is the left hemisphere [9,21, 26]. Furthermore, exhaustive psychosocial and repeated psychiatric assessments of the patient revealed nothing in his pre- or post-morbid history to suggest a psychogenic contribution to his amnesic condition. Five neurologists, two neurosurgeons, two psychiatrists, and three psychologists examined DD over a 15-year period. They all carefully considered and finally rejected the psychogenic hypothesis on the basis of his background and clinical profile. Moreover, DD exhibits only 4 of 23 features considered by Kapur [16] to support a psychological basis to a retrograde amnesia. Those features are : 1. disproportionate severity in regard to lesion, 2. absence of diffuse brain pathology, 3. loss of basic personal semantic knowledge, and 4. failure to recognise as familiar, members of his family. The fact that DD did not recognize as familiar, pictures of himself at a younger age, nor pictures of his family, may indeed appear psychogenic-like since it is usually assumed that the loss of self-sense does not happen in organic amnesia. It must be noted that Kapur's features do not include more recent, post-1999 cases that do not conform to this characterisation [31]. An interesting and genuine counter-example of this argument may be found in a paper from Meltzer [30]. The author, a psychologist, suffered a cardiac arrest which resulted in anoxia and partial retrograde amnesia, a loss of past memories and the feeling of not quite exactly knowing anymore who he was. This partial loss (or more complete loss as in the case of DD) in the case of anoxia, could lead to difficulties such as not recognizing oneself in old pictures or not being able to answer some questions about personal values, as was the case for DD when he completed the MMPI.

To our knowledge, one other patient has been reported with an extensive and isolated retrograde amnesia following a circumscribed thalamic lesion. JG, a 33-year old man, showed a dense, remote memory impairment after having suffered a likely infarction in the right thalamus [31]. MRI images yielded evidence of a more recent thalamic lesion involving the right medial region and an older one involving bilateral posteromedial regions. Psychiatric investigation revealed no evidence of a functional aetiology or malingering. From a clinical point of view, it is of particular interest that JG showed a reverse temporal gradient and a loss of some aspects of his prior procedural knowledge, both features being quite rare in retrograde amnesia, in addition to a loss of some semantic knowledge. The authors concluded on the crucial role for the right mesiodorsal region of the thalamus in the ability to access remote memories.

It is very unlikely that DD could be malingering, especially in the face of his unchanged, twenty-six year-long state of amnesia. Also, there was no evidence of secondary gains. If there was at attempt for emotional or financial compensation for his dysfunction, he would not likely have gone back to work with such effort and persistence only a few months after his accident. Furthermore, DD went on to live a normal and autonomous widower's life, after his wife's death in 1993, an attitude that does not seem typical of a person who would have been simulating amnesia in order to escape responsibilities. Our tentative hypothesis to explain DD's invasive amnesia in the presence of such a focal brain lesion is that the latter may have disrupted the structures and mechanisms devoted to the retrieval and reconstruction of autobiographical memories. It remains to be determined if there are specific regions specialised in the retrieval of remote souvenirs and if so, their localization. A role for the thalamus in coordinating cortical networks involved in conscious recollection has been put forward [2,24].

We believe that an interesting way to promote a better understanding of FRA would be to extend the approach described in this paper and directly compare patients with FRA of organic and psychogenic origin, using functional imaging soon after the onset of the amnesia, in order to control the relearning of personal memories.

## Aknowledgement

We wish to thank the patients AC and DD, and their relatives, for their collaboration.
